# Supplementary material for: Anemia in Celiac Disease: Prevalence, Associated Clinical and Laboratory Features, and Persistence after Gluten-Free Diet
Source: J Pers Med. 2022 Sep 26;12(10):1582. doi: 10.3390/jpm12101582 (PMC9604793; doi:10.3390/jpm12101582)
Supplement: Supplementary file 1 [file jpm-12-01582-s001.zip › jpm-1880603-supplementary 1.pdf]

**Supplemental File 1. Reference values of the hematochemical parameters reported.**

| | **Reference values** |
|---|---|
| Hemoglobin (HGB) | |
| Male | 13-18 g/dL |
| Female | 12-16 g/dL |
| Hematocrit (HCT) | 37-52% |
| Mean Corpuscular Volume (MCV) | 80-99 fL |
| Mean Corpuscular HGB (MCH) | 26-32 pg |
| Mean Corpuscular HGB Concentration (MCHC) | 32-36 g/dL |
| Red Cell Distribution Width (RDW) | 11-15% |
| Total Serum Iron | |
| Male | 65-180 μg/dL |
| Female | 30-170 μg/dL |
| Ferritin | 15-150 ng/mL |
| Transferrin | 204-360 mg/dL |
| Erythrocyte Sedimentation Rate (ESR) (1st. hour) | 2-20 mm/h |
| C-Reactive Protein (CRP) | <5 mg/l |
| Vitamin $B_{12}$ | 197-890 ng/L |
| Folic acid | 3.89-26.8 mcg/L |
| Thyroid-Stimulating Hormone (TSH) | 0.35-4.94 μU/mL |
| Anti-Nuclear Antibodies (ANA) | Negative |
