# Supplementary material for: Anemia in Celiac Disease: Prevalence, Associated Clinical and Laboratory Features, and Persistence after Gluten-Free Diet
Source: J Pers Med. 2022 Sep 26;12(10):1582. doi: 10.3390/jpm12101582 (PMC9604793; doi:10.3390/jpm12101582)
Supplement: Supplementary file 1 [file jpm-12-01582-s001.zip › jpm-1880603-supplementary 2.pdf]

Table S2.1. Demographic, clinical, histological and serological features of CD patients.

	**CD (n=159)**
Sex	
Female	129 (81.1%)
Male	30 (18.9%)
Age at diagnosis (years) (mean ± SD)	35.4 ± 14.7
Diagnostic delay (months) (median ± range)	36.0 (1-732)
BMI (mean ± SD)	22.9 ± 4.1
IBS-like symptoms	
None	32 (20.1%)
Diarrhea	83 (52.2%)
Constipation	24 (15.1%)
Alternating bowel movements	20 (12.6%)
Dyspepsia	67 (42.1%)
Extra-intestinal symptoms	108 (67.9%)
Weight loss	55 (34.6%)
Poly/hypermenorrhea	39 (24.5%)
Associated autoimmune diseases	55 (34.6%)
MFH	12 (7.5%)
Nickel hypersensitivity	15 (9.4%)
SRMI	48 (30.2%)
Atopy	26 (16.4%)
Marsh duodenal histology	
2	7 (4.4%)
3	152 (95.6%)

BMI=Body Mass Index; CD=Celiac Disease; IBS=Irritable Bowel Syndrome; MFH=Multiple Food Hypersensitivity; SD=Standard Deviation; SRMI=Self-Reported Milk Intolerance.

Table S2.2. Frequency of extra-intestinal symptoms referred in the whole CD population.

	CD (n=159) (%)
Skin disorders (1 or more)	24 (15.0)
Erythema and/or eczema	8 (5.0)
Itching	7 (4.4)
Urticaria-angioedema	7 (4.4)
Alopecia	4 (2.5)
Nail fragility	2 (1.3)
Oral mucosa disorders (1 or more)	19 (11.9)
Recurrent aphthous stomatitis	17 (10.7)
Angular cheilitis	2 (1.3)
Skeletal system (1 or more)	26 (16.3)
Arthralgia	19 (11.9)
Myalgia	13 (8.1)
Neuropsychiatric symptoms (1 or more)	56 (35.2)
Headache/migraine	30 (18.9)
Asthenia	27 (17.0)
Sensory disorders (Numbness, paresthesia, dysesthesia)	7 (4.4)
Anxiety/ depression	6 (3.8)
Confusion/mental fog	6 (3,8)
Insomnia, diurnal drowsiness	4 (2.5)
Visual disorders	3 (1.9)
Attention or concentration deficit	2 (1.3)
Lipothymia/ syncope	2 (1.3)
Vertigo or other balance disorders	1 (0.6)
Cardiac symptoms (1 or more)	
Palpitations/tachycardia/ectopic beats	6 (3.8)
Genitourinary disorders (1 or more)	
Cystitis, dysmenorrhea, dyspareunia	13 (8.2)

CD=Celiac Disease

Table S2.3. Frequency of autoimmune diseases referred in the whole CD population.

	CD (n=159) (%)
Hashimoto's thyroiditis	27 (17.0)
Psoriasis ± psoriatic arthritis	5 (3.1)
Raynaud's phenomenon	5 (3.1)
Fibromyalgia	4 (2.5)
Diabetes mellitus (Type 1)	4 (2.5)
Vitiligo	3 (1.9)
Autoimmune hepatitis	2 (1.3)
Autoimmune atrophic gastritis	2 (1.3)
Graves' disease	2 (1.3)
Multiple sclerosis	2 (1.3)
Sjogren's syndrome	2 (1.3)
Undifferentiated connectivitis	2 (1.3)
Ankylosing spondylitis	1 (0.6)
Pemphigus foliaceus	1 (0.6)
Rheumatoid arthritis	1 (0.6)
Seronegative spondyloarthritis	1 (0.6)

CD=Celiac Disease

Table S2.4. Blood count in 159 CD patients.

	CD (n=159)
HGB (gr/dl) (mean ± SD)	11.9 ± 2.1
Hematocrit	
Below normal value (number and %)	54 (34.0)
Hematocrit (%) (mean ± SD)	37.9 ± 4.5
MCV	
Below normal value (number and %)	85 (53.5)
MCV (fL) (mean ± SD)	81.2 ± 9.1
MCH	
Below normal value (number and %)	77 (48.4)
MCH (pg) (mean ± SD)	26.9 ± 2.9
MCHC	
Below normal value (number and %)	29 (18.2)
MCHC (gr/dl) (mean ± SD)	32.8 ± 1.7
RDW	
Above normal value (number and %)	24 (15.1)
RDW (%) (mean ± SD)	14.5 ± 2.5

CD=Celiac Disease; HGB=Hemoglobin; MCH=Mean Corpuscular HGB; MCHC=Mean Corpuscular HGB Concentration; MCV=Mean Corpuscular Volume; RDW=Red Cell Distribution Width; SD=Standard Deviation.

References values: Hemoglobin (HGB) Male 13-18 g/dL, Female 12-16 g/dL; Mean Corpuscular HGB (MCH) 26-32 pg; Mean Corpuscular HGB Concentration (MCHC) 32-36 g/dL; Mean Corpuscular Volume (MCV) 80-99 fL; Red Cell Distribution Width (RDW) 11-15%.

Table S2.5. Iron metabolism, ESR, CRP, vitamin B_{12}, folic acid and TSH in 159 CD patients.

	CD (n=159)
Total serum iron	
Below normal value (number and %)	79 (49.7)
Total serum iron (μg/dL) [median (range)]	47.5 (18-157)
Serum ferritin	
Below normal value (number and %)	101 (63.5)
Above normal value (number and %)	6 (3.8)
Serum ferritin (ng/ml) [median (range)]	13.2 (2-284)
ESR	
Above normal value (number and %)	68 (42.8)
ESR (mm/h) [median (range)]	22 (2-75)
CRP	
Above normal value (number and %)	17 (10.7)
CRP (mg/dl) [median (range)]	0.62 (0.06-32)
Vitamin B_{12}	
Below normal value (number and %)	49 (30.8)
Vitamin B_{12} (pg/mL) [median (range)]	605 (113-903)
Folic acid	
Below normal value (number and %)	76 (47.8)
Folic acid (ng/mL) [median (range)]	2.34 (0.6-11.5)
TSH	
Above normal value (number and %)	37 (23.3)
TSH (microIU/l) (mean ± SD)	1.9 ± 0.8

CD=Celiac Disease; CRP=C-Reactive Protein; ESR=Erythrocyte Sedimentation Rate; SD=Standard Deviation; TSH=Thyroid-Stimulating Hormone.

Reference values: C-reactive protein (CRP) <5 mg/l; Erythrocyte Sedimentation Rate (ESR) 2-20 mm/h; Ferritin 15-150 ng/mL; Folic acid 3.89-26.8 mcg/L; Thyroid-Stimulating Hormone (TSH) 0.35-4.94 μU/mL; Total Serum Iron Male 65-180 μg/dL, Female 30-170 μg/dL; Vitamin B_{12} 197-890 ng/L.
