# Supplementary figures and images for: Anemia in Celiac Disease: Prevalence, Associated Clinical and Laboratory Features, and Persistence after Gluten-Free Diet
Source: J Pers Med. 2022 Sep 26;12(10):1582. doi: 10.3390/jpm12101582 (PMC9604793; doi:10.3390/jpm12101582)

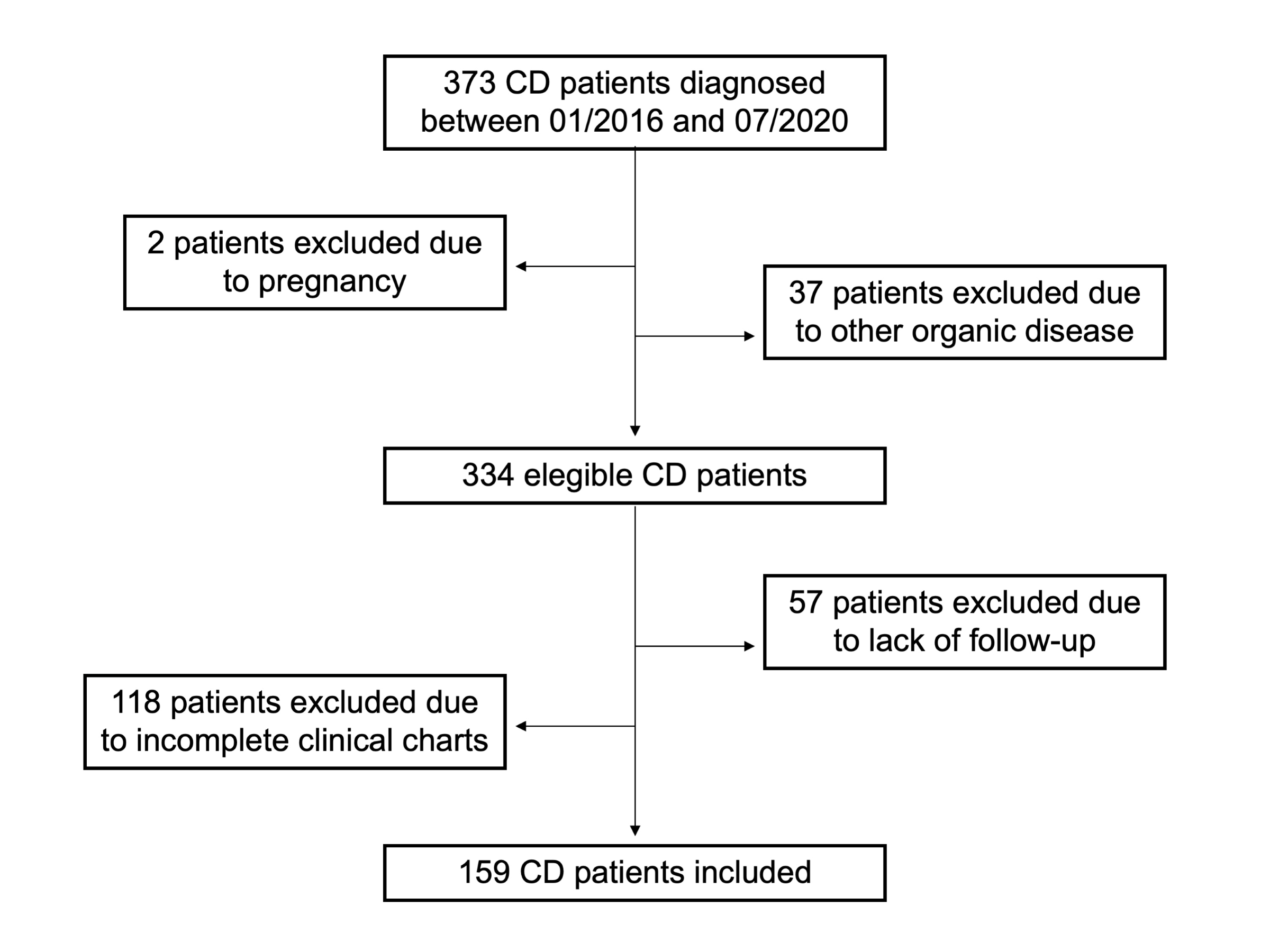

Supplement: Supplementary file 1 [file jpm-12-01582-s001.zip › Supplementary Figure 1 07.08.jpeg]
